# Disulfide-Crosslinked mPEG-PLA-(LA)_4_ Nanomicelles for Taxane Delivery in Breast Cancer Therapy

**DOI:** 10.3390/molecules31132238

**Published:** 2026-06-25

**Authors:** Yukun Xie, Hao Wang, Lanlan Xiang, Yuchen Shen, Yujie Yang, Jiajie Liu, Xin Teng

**Affiliations:** 1 Shanghai Key Laboratory of Advanced Polymeric Materials, School of Materials Science and Engineering, East China University of Science and Technology, 130 Meilong Road, Shanghai 200237, China; xieyukun@mail.ecust.edu.cn (Y.X.);; 2 Faculty of Biomedical Engineering, University of New South Wales, Sydney, NSW 2052, Australia; 3 International Elite Engineering School, East China University of Science and Technology, Shanghai 200237, China

**Keywords:** paclitaxel, docetaxel, cabazitaxel, nanomicelles, breast cancer, disulfide-crosslinked

## Abstract

Taxanes represent a crucial class of chemotherapeutic agents clinically employed for the treatment of various malignancies, including breast cancer. Three commonly utilized taxanes—paclitaxel (Taxol), docetaxel (Taxotere), and cabazitaxel—are substantially limited by inherent drawbacks such as poor aqueous solubility, rapid clearance, and non-specific distribution. To enhance their anti-tumor efficacy against breast cancer, an mPEG-PLA-(LA)_4_ nanomicelle carrier was designed and synthesized in this study for the loading of these three taxanes. These nanomicelles were designed to improve the aqueous compatibility and nanomicelle-mediated delivery of hydrophobic taxanes. In vivo animal experiments were conducted to evaluate the therapeutic effects of the three drug-loaded nanomicelles on subcutaneous human breast cancer MCF-7 xenografts in BALB/c nude mice. The results demonstrated that the injectable paclitaxel, docetaxel, and cabazitaxel micelles exhibited significant inhibitory effects on the MCF-7 xenograft tumors. These findings suggest that mPEG-PLA-(LA)_4_ DS nanomicelles may serve as a structurally defined and versatile carrier platform for hydrophobic taxane delivery.

## 1. Introduction

According to the International Agency for Research on Cancer (IARC) of the World Health Organization (WHO), if current epidemiological trends continue, the global burden of breast cancer will exacerbate significantly by 2050. Over the next 25 years, the number of new breast cancer cases worldwide is projected to increase by approximately 38%, and mortality will rise by roughly 68%. By then, annual incident cases will exceed 3.2 million, with annual deaths approaching 1.1 million. Concurrently, the lifetime risk of developing breast cancer continues to escalate, with an estimated 1 in 20 women likely to be diagnosed with the disease during their lifetime. Notably, this growth is unevenly distributed geographically; Asia, with particularly populous nations such as China, will be the primary contributor to this future increase, demonstrating a remarkably pronounced upward trend in the disease burden [1]. Chemotherapy, encompassing both adjuvant and neoadjuvant approaches, can effectively mitigate the risk of recurrence in patients with early-stage cancer [2]. Taxanes constitute a vital class of chemotherapeutic agents utilized clinically for various malignancies, including breast cancer, with paclitaxel, docetaxel, and cabazitaxel being the most administered.

As one of the most potent classes of anticancer agents, taxanes, initially isolated from the bark of the Pacific yew (*Taxus brevifolia*), are widely employed in the treatment of various malignancies, including lung, breast, and ovarian cancers [3]. As prototypical microtubule stabilizers, taxanes primarily bind to the β-subunit of tubulin with high affinity, promoting the aberrant assembly of tubulin monomers and profoundly inhibiting microtubule depolymerization. This hyper-stabilization disrupts normal mitotic spindle formation, triggering the spindle assembly checkpoint (SAC) to irreversibly arrest the cell cycle at the late G2 or M phase, ultimately inducing apoptosis [4]. Furthermore, recent studies have elucidated a highly selective “second mechanism” whereby taxane-induced rigid microtubule bundles exert physical traction on the fragile nuclear envelope of cancer cells during interphase, causing nuclear envelope rupture and micronucleation [5,6]. Concurrently, these drugs actively modulate intracellular apoptotic pathways by downregulating the anti-apoptotic protein Bcl-2 [7]. Despite these robust mechanisms, the clinical application of paclitaxel, docetaxel, and cabazitaxel is severely hindered [8] by their limited aqueous solubility, propensity for recrystallization upon dilution, rapid blood clearance, non-specific distribution [9], and cosolvent-induced toxicity [10].

The clinical application of taxanes is severely limited by their extremely poor aqueous solubility, rapid blood clearance, non-specific systemic distribution, and limited tumor accumulation [11]. To overcome this profound physical limitation, commercial paclitaxel formulations are typically prepared with a solvent system comprising Cremophor EL (polyethoxylated castor oil) and dehydrated ethanol, whereas docetaxel (DTX) and cabazitaxel (CTX) rely on Tween-80 as a solubilizer. However, these conventional solubilizers elicit an exceptionally high incidence of clinical adverse reactions. Clinical data indicate that Cremophor EL induces severe hypersensitivity reactions in up to 30% of unpremedicated patients; even with routine premedication using steroids and antihistamines, 2–4% of patients still experience life-threatening severe allergic reactions, compelling the discontinuation of therapy [12]. Similarly, in addition to causing allergic symptoms such as dyspnea, bronchospasm, and hypotension [13,14], Tween-80 leads to a pronounced cumulative fluid retention syndrome in over 50% of unpremedicated patients, forcing a substantial number of patients to interrupt chemotherapy due to pleural and peritoneal effusions or severe edema [15]. The allergic reactions induced by DTX may be mediated by type I immunoglobulin E (IgE) responses or caused by the direct action of the drug on mast cells and basophils, resulting in a massive release of histamine [16,17]. To prevent and mitigate these allergic reactions, premedication with dexamethasone is required prior to DTX infusion. Nevertheless, the extensive side effects of dexamethasone significantly restrict its utility; it can induce diabetes, hypertension, and hyperlipidemia, severely compromising the cardiovascular function of patients and elevating the mortality risk associated with cardiovascular events [18,19].

Cabazitaxel (CTX), a semi-synthetic tubulin-binding taxane derivative, was developed as a second-generation taxane for clinical application [20]. Unlike docetaxel, CTX exhibits a low substrate affinity for P-glycoprotein (P-gp); consequently, it is not effluxed by the P-gp pump following cellular uptake [21], which endows cabazitaxel with a superior capacity to overcome drug resistance. Similarly to other taxanes, cabazitaxel exerts its pharmacological effects by binding to microtubules, thereby arresting cellular mitosis and suppressing tumor cell proliferation [22]. Its commercial formulation, Jevtana^®^, was approved by the United States Food and Drug Administration (FDA) in 2010 for the treatment of patients with refractory metastatic prostate cancer [23].

Nanocarrier-based drug delivery systems have been developed to improve the solubility, stability, and pharmacological behavior of poorly water-soluble anticancer drugs. Consequently, driven by advancements in tumor diagnosis and treatment, smarter third-generation nanocarriers with enhanced theranostic value have been introduced [24]. Accompanying this progression, various novel formulations have emerged, such as nanoparticles [25,26], liposomes [27,28], nanofibers [29], and nanomicelles [30,31]. Particularly, micellar formulations of diverse antineoplastic agents have been extensively investigated in both preclinical and clinical trials. Polymeric micelles, characterized by their unique core–shell structure [32], can significantly enhance the solubility, stability, and delivery performance of poorly water-soluble compounds [33,34]. By utilizing nanomicelles as delivery carriers for paclitaxel and docetaxel, the severe allergic reactions induced by conventional solubilizers (Cremophor EL and Tween-80) can be entirely circumvented, while potentially improving antitumor efficacy.

LA-containing and disulfide-crosslinked micelles have been previously investigated for redox-responsive taxane delivery. For example, Han et al. reported cabazitaxel-loaded disulfide-crosslinked micelles prepared from lipoic-acid-grafted mPEG-PLA, demonstrating the feasibility of LA-mediated core crosslinking for CTX delivery [35]. Li et al. developed reversible disulfide-crosslinked micelles for on-demand paclitaxel delivery, representing a classic disulfide-crosslinked taxane micelle system [36]. Therefore, the present study does not claim the first use of LA-crosslinked or disulfide-crosslinked micelles, but focuses on a structurally defined mPEG-PLA-(LA)_4_ carrier bearing four predetermined LA moieties and its application to taxane delivery. In this study, we developed an mPEG-PLA-(LA)_4_ nanomicelle carrier designed to encapsulate these three taxanes. Furthermore, we report the in vivo outcomes of evaluating these nanomicelles as taxane delivery carriers in MCF-7 breast cancer xenograft models. Our results demonstrate that the injectable paclitaxel, docetaxel, and cabazitaxel micelles exhibit pronounced inhibitory effects on human breast cancer MCF-7 subcutaneous xenografts in nude mice. In addition to the structurally defined (LA)_4_ carrier architecture, another objective of this study was to evaluate whether the same mPEG-PLA-(LA)_4_ DS micelle platform could be applied to multiple clinically relevant taxanes. Representative redox-responsive taxane micelles reported previously usually focus on one taxane per formulation, such as PTX-, DTX- or CTX-loaded micelles. Therefore, the present study applied the same carrier platform to PTX, DTX and CTX to assess its formulation versatility for hydrophobic taxane delivery.

## 2. Results and Discussion

### 2.1. Synthesis, Defined (LA)_4_ Architecture, and Structural Characterization of mPEG-PLA-(LA)_4_ Nanocarrier

**Scheme 1:**
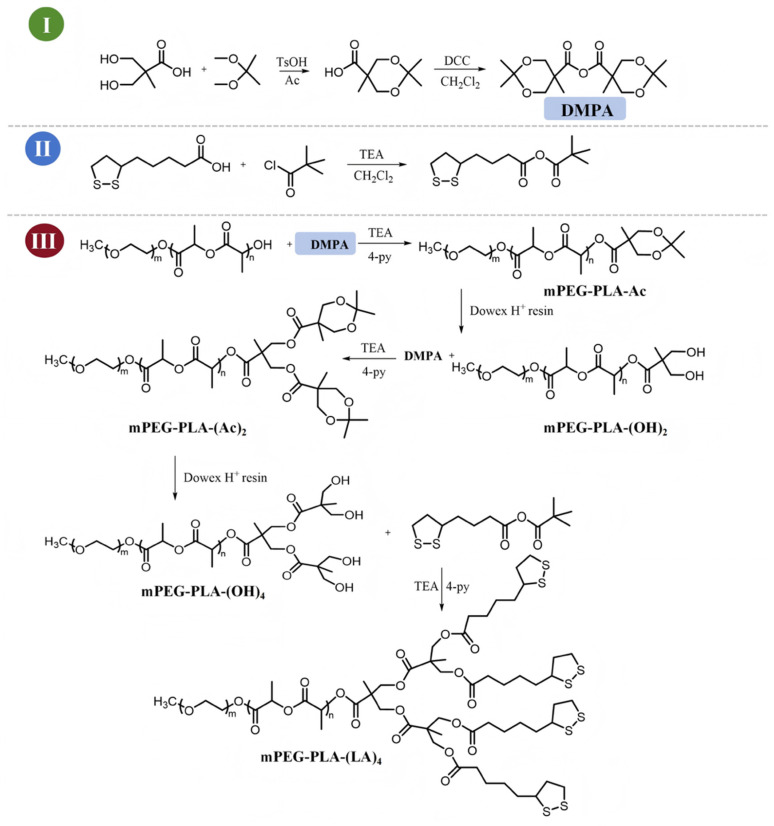
Synthetic route of structurally defined mPEG-PLA-(LA)_4_ copolymer bearing four predetermined LA moieties as fixed disulfide-forming sites.

The synthetic route for the disulfide-crosslinked mPEG-PLA-(LA)_4_ block copolymer is illustrated in Scheme 1. To verify the successful synthesis of the target material, FT-IR spectroscopy, Raman spectroscopy, and ^1^H-NMR spectroscopy were employed for structural characterization.

**Figure 1:**
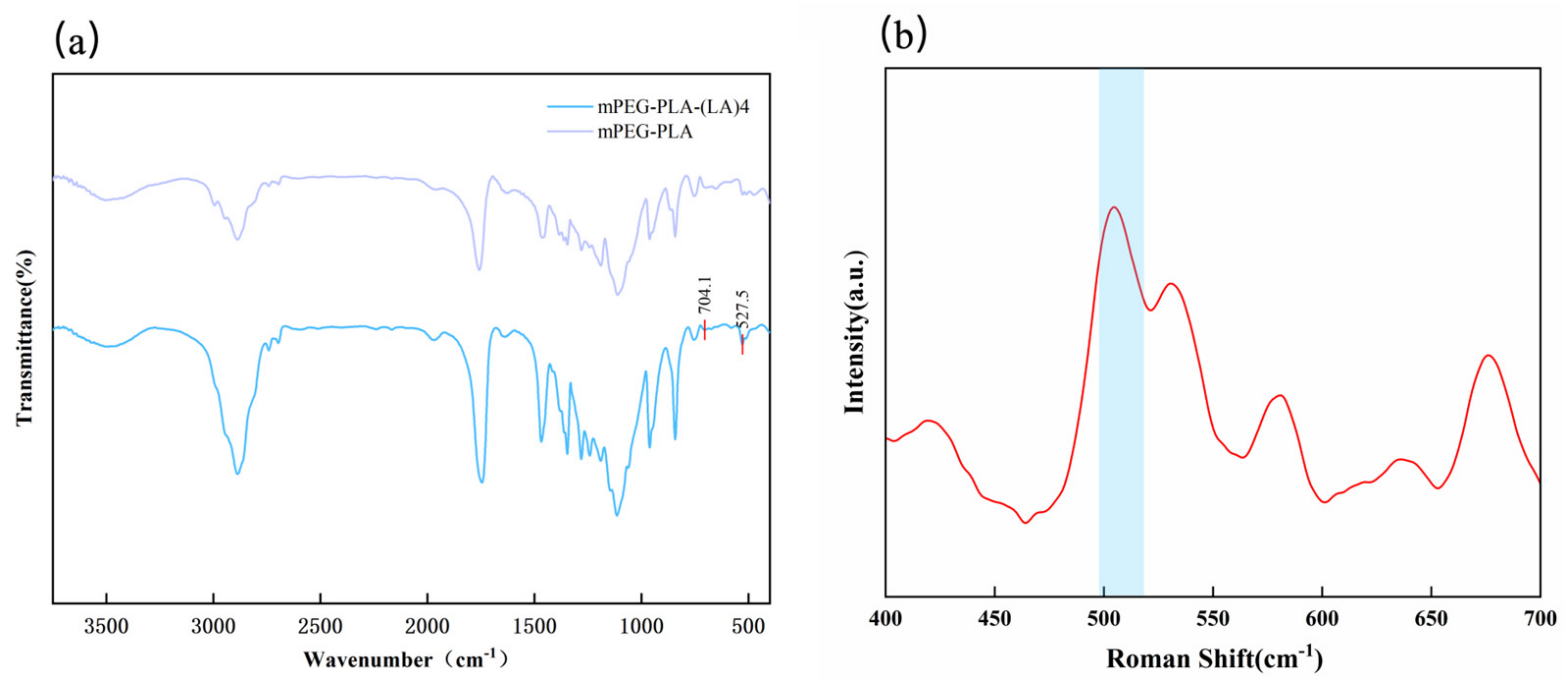
(**a**) FT-IR spectra of mPEG-PLA and mPEG-PLA-(LA)_4_. (**b**) Raman spectra of mPEG-PLA and mPEG-PLA-(LA)_4_ in the characteristic S–S vibration region.

The FT-IR spectra of mPEG-PLA-(LA)_4_ and its precursor are presented in Figure 1a. Compared with mPEG-PLA, mPEG-PLA-(LA)_4_ exhibited new absorption bands at 704 cm^−1^ and 528 cm^−1^, which are consistent with LA-derived C–S and S–S vibrations, respectively. The appearance of the C–S absorption band indicates the successful introduction of LA-containing structures into the mPEG-PLA copolymer. However, because the S–S stretching vibration is generally weak in FT-IR, Raman spectroscopy was further performed to provide complementary evidence for the disulfide bond. As shown in Figure 1b, mPEG-PLA-(LA)_4_ displayed a Raman band at approximately 510 cm^−1^ within the characteristic 500–550 cm^−1^ region of disulfide S–S stretching vibrations. Taken together, the FT-IR and Raman results support the successful introduction of LA-derived disulfide-containing structures into the mPEG-PLA copolymer.

**Figure 2:**
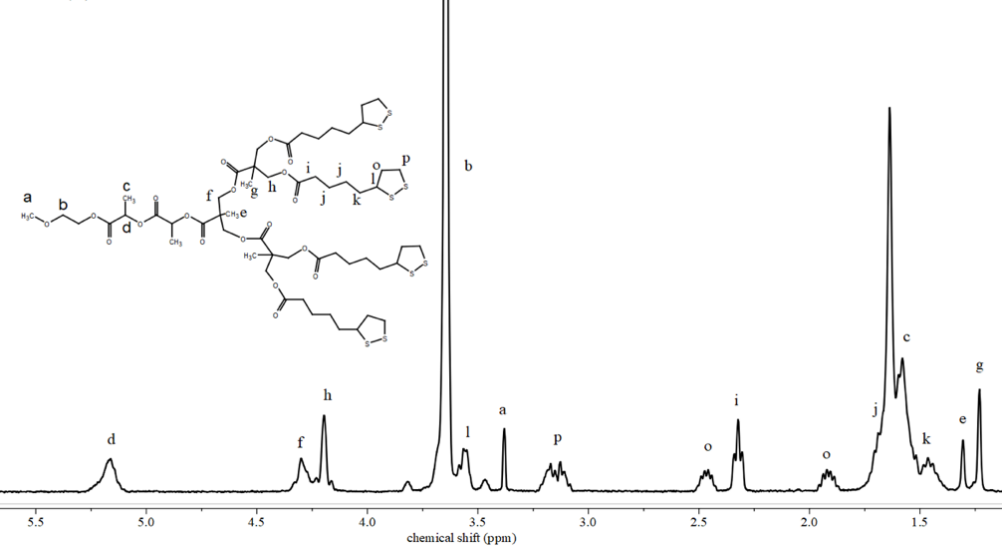
^1^H-NMR spectrum of mPEG-PLA-(LA)_4_.

The ^1^H-NMR spectrum of mPEG-PLA-(LA)_4_ is shown in Figure 2. As illustrated, the chemical shifts at δ = 2.32 ppm (i), δ = 1.69 ppm (j), δ = 1.46 ppm (k), δ = 3.55 ppm (l), δ = 1.92/2.46 ppm (o), and δ = 3.13 ppm (p) were all identified as the characteristic absorption peaks of the LA moieties. Furthermore, the integration area ratios of these corresponding peaks were consistent with the theoretical number of hydrogen atoms, following the relationships: 2o = p = i and 2e = g. Meanwhile, the integration ratios among the original backbone peaks remained unchanged. Collectively, these spectral results support that four LA molecules were successfully grafted onto the mPEG-PLA backbone, confirming the successful synthesis of the mPEG-PLA-(LA)_4_ copolymer.

Importantly, the present mPEG-PLA-(LA)_4_ copolymer possesses a structurally defined tetravalent LA architecture. In this design, the DMPA-derived branching unit provides four terminal reactive sites, which are subsequently conjugated with LA to generate four predetermined disulfide-forming points. This structure is different from previously reported LA-grafted or disulfide-crosslinked redox-responsive micellar systems, in which LA-containing or disulfide-forming units are not necessarily presented as a fixed tetravalent terminal architecture. Such fixed LA sites may provide more controllable intramicellar crosslinking and reduce structural heterogeneity during micelle formation. The FT-IR and Raman results confirm the introduction of LA-derived C–S and S–S signals, while the ^1^H-NMR integration ratios further support the successful grafting of four LA moieties onto the mPEG-PLA backbone. Therefore, these structural-characterization results provide molecular-level evidence for the designed mPEG-PLA-(LA)_4_ nanocarrier.

### 2.2. Physicochemical Characterization of CTX-Loaded Nanomicelles Prepared at Different Theoretical Drug Loading Ratios

**Figure 3:**
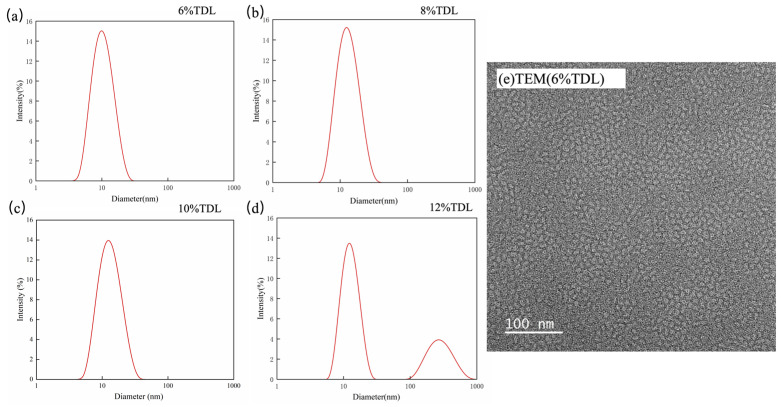
Particle size distribution and representative TEM morphology of CTX-loaded disulfide-crosslinked nanomicelles. DLS intensity-based size distributions of CTX-loaded nanomicelles prepared at theoretical drug loading ratios of (**a**) 6.0%, (**b**) 8.0%, (**c**) 10.0%, and (**d**) 12.0%. (**e**) Representative TEM image of CTX-loaded disulfide-crosslinked nanomicelles at 6.0% theoretical drug loading. Scale bar = 100 nm.

Dynamic light scattering (DLS) was employed to characterize the hydrodynamic diameter and size distribution of CTX-loaded disulfide-crosslinked nanomicelles prepared at different theoretical drug loading ratios (TDL). As shown in Figure 3a–d and summarized in Table 1, the nanomicelles prepared at 6.0%, 8.0%, and 10.0% TDL exhibited narrow size distributions, with hydrodynamic diameters of 10.15 ± 0.02 nm, 12.36 ± 0.03 nm, and 12.75 ± 0.02 nm, respectively. The corresponding PDI values were 0.194 ± 0.001, 0.158 ± 0.011, and 0.211 ± 0.004, indicating acceptable colloidal uniformity. When the TDL was further increased to 12.0%, the PDI increased markedly to 0.460 ± 0.008, and the DLS profile displayed a bimodal distribution, suggesting reduced colloidal uniformity at the higher feeding ratio. Therefore, 10.0% TDL was selected as the working formulation ratio for subsequent experiments because it provided a relatively high nominal drug input while maintaining a small hydrodynamic diameter and acceptable PDI.

In addition to hydrodynamic diameter and PDI, zeta potential was measured to evaluate the surface charge characteristics of the CTX-loaded disulfide-crosslinked nanomicelles. As summarized in Table 1, the formulations exhibited near-neutral to slightly negative zeta potentials, which is consistent with the PEG-shielded surface of the micelles. These results suggest that increasing the theoretical drug feeding ratio did not markedly disturb the surface charge characteristics of the nanomicelles.

To complement the DLS characterization with real-space morphological information, TEM observation was performed on representative CTX-loaded disulfide-crosslinked nanomicelles prepared using the same formulation procedure. As shown in Figure 3e, the nanomicelles displayed nanosized, approximately spherical dot-like morphology without obvious large aggregates. Together, the DLS, zeta-potential, and TEM results support the formation of nanosized micellar assemblies.

The particle-size comparison with representative disulfide-crosslinked taxane micellar systems is summarized in Table 2. The optimized CTX-loaded mPEG-PLA-(LA)_4_ DS nanomicelles prepared at 10.0% TDL showed an ultrafine hydrodynamic diameter of 12.75 ± 0.02 nm with an acceptable PDI of 0.211 ± 0.004. The apparent encapsulation efficiency of the optimized 10.0% TDL formulation was further evaluated using a centrifugation–UV spectrophotometric method. At 228 nm, the absorbance of the post-centrifugation supernatant was 1.060, whereas the matched LA4 carrier blank showed an absorbance of 0.283. After correction for carrier-derived absorption, the CTX concentration was determined to be 39.67 μg/mL, compared with the theoretical concentration of 40.0 μg/mL. Accordingly, the apparent encapsulation efficiency was 99.18%, indicating that most of the initially added CTX remained associated with the nanomicellar formulation after centrifugation. Such a small size may be beneficial for tumor interstitial diffusion, as Cabral et al. reported that 30 nm polymeric micelles penetrated poorly permeable tumors more efficiently than larger 50–100 nm micelles [37]. In addition, Chauhan et al. suggested that smaller nanomedicines of approximately 12 nm exhibit superior tumor penetration [38]. Nevertheless, direct tumor-penetration and biodistribution experiments were not performed in the present study; therefore, this size-related advantage should be interpreted as a literature-supported possibility rather than direct experimental proof.

### 2.3. In Vivo Antitumor Efficacy and Preliminary Tolerability Evaluation

#### 2.3.1. Body Weight Changes

**Figure 4:**
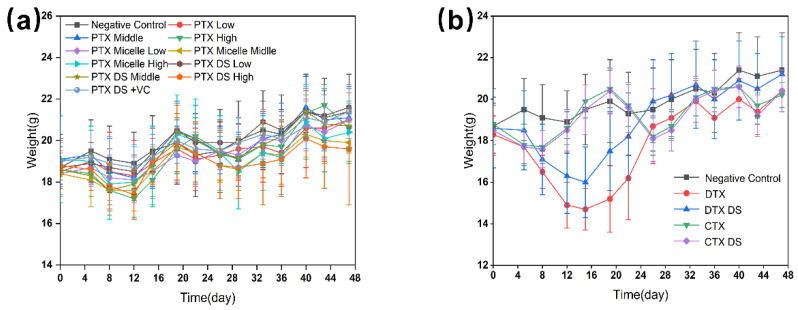
Body weight changes of BALB/c nude mice bearing MCF-7 tumor xenografts after different treatments over time. (**a**) Paclitaxel (PTX) formulations with different doses; (**b**) Docetaxel (DTX) and cabazitaxel (CTX) formulations.

Before administration, the body weights of the animals across all groups were similar. As depicted in Figure 4a, the body weights of the negative control group generally exhibited an upward trend following administration, reaching 21.4 ± 1.8 g on day 47 (D47).

From post-administration to D47, no statistically significant differences (*p* > 0.05) were observed in the body weights of the low- and middle-dose albumin-bound paclitaxel groups, the low- and high-dose injectable paclitaxel micelle groups, the low-dose injectable paclitaxel DS micelle group, and the low-dose injectable paclitaxel DS micelle + VC group compared to the negative control group. Their respective weights on D47 were 21.0 ± 1.6 g, 21.1 ± 1.4 g, 21.0 ± 1.3 g, 20.4 ± 1.4 g, 21.6 ± 1.0 g, and 21.4 ± 1.3 g.

Conversely, the body weights of mice in the high-dose albumin-bound paclitaxel group were significantly lower than those of the negative control group between D8 and D15 (*p* ≤ 0.05 and *p* ≤ 0.01), with a weight of 20.6 ± 1.0 g on D47. The body weights in the middle-dose injectable paclitaxel micelle group, the middle-dose injectable paclitaxel DS micelle group, and the high-dose injectable paclitaxel DS micelle group were significantly lower than the negative control group during the periods of D8–D12, D8, and D8–D12, respectively (*p* ≤ 0.05), with weights recording 19.9 ± 1.0 g, 20.7 ± 1.2 g, and 19.6 ± 2.7 g on D47.

As shown in Figure 4b, the body weights of the docetaxel injection group were significantly lower than the negative control group from D8 to D22 (*p* ≤ 0.001), recovering to 20.4 ± 1.0 g on D47. The body weights of the injectable docetaxel DS micelle group were significantly lower than the negative control group from D8 to D19 (*p* ≤ 0.01 and *p* ≤ 0.001); however, the weights in this group were consistently higher than those in the docetaxel injection group post-administration, with significant differences observed on D12 and between D19–D22 (*p* ≤ 0.05, *p* ≤ 0.01), reaching 21.2 ± 1.8 g on D47. The body weights in both the cabazitaxel injection group and the injectable cabazitaxel DS micelle group were significantly lower than the negative control group on D8 (*p* ≤ 0.05), ending at 20.2 ± 0.6 g and 20.4 ± 1.0 g on D47, respectively.

#### 2.3.2. Changes in Tumor Volume and Relative Tumor Volume (RTV)

**Figure 5:**
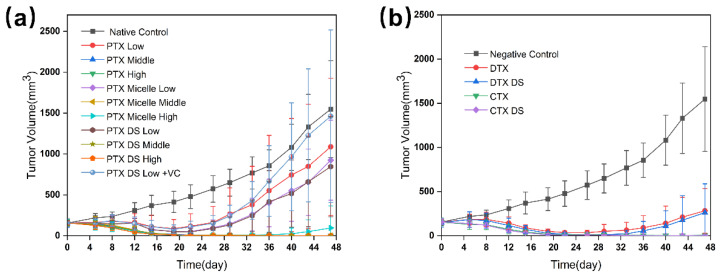
Tumor volume changes of MCF-7 tumor-bearing mice over time following respective intravenous administrations. (**a**) Paclitaxel formulations; (**b**) Docetaxel and cabazitaxel formulations.

**Figure 6:**
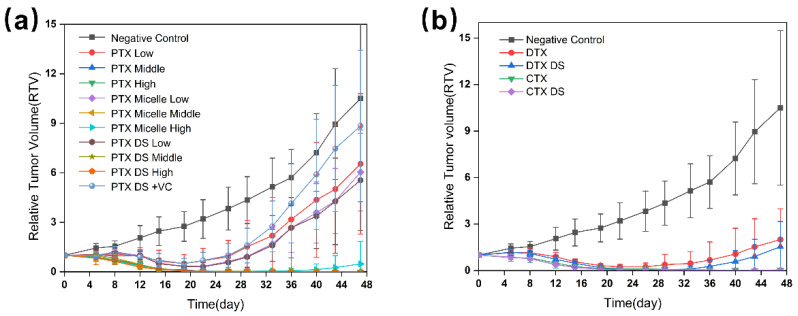
Changes in relative tumor volume (RTV) of MCF-7 tumor-bearing mice across all treatment groups. (**a**) Paclitaxel formulations; (**b**) Docetaxel and cabazitaxel formulations.

As illustrated in Figure 5 and Figure 6, the initial tumor volumes before the first administration were similar across all groups, averaging 154.84 ± 34.42 mm^3^. Following administration, the tumor volume in the negative control group grew continuously, reaching a mean volume of 1547.86 ± 592.34 mm^3^ and a mean relative tumor volume (RTV) of 10.50 ± 4.98 on D47.

The low-, middle-, and high-dose albumin-bound paclitaxel groups all exhibited varying degrees of tumor growth inhibition. For the middle-dose group, the tumor volume and RTV were significantly lower than those of the negative control group from D8 to D47 and D5 to D47, respectively (*p* ≤ 0.001), with the tumor volume shrinking to 0.85 ± 2.41 mm^3^ and an RTV of 0.00 ± 0.01 on D47. The high-dose group achieved complete tumor regression, with both tumor volume and RTV recording 0.00 ± 0.00 on D47.

The low-, middle-, and high-dose injectable paclitaxel micelle groups also exhibited varying degrees of tumor inhibition post-administration. In the low-dose group, the tumor volume and mean RTV were significantly lower than those of the negative control group from D12 to D47 and D5 to D47, respectively (*p* ≤ 0.05, *p* ≤ 0.01, and *p* ≤ 0.001), resulting in a tumor volume of 923.72 ± 489.26 mm^3^ and an RTV of 6.04 ± 2.36 on D47. The middle-dose group showed significantly reduced tumor volume and RTV compared to the negative control group from D8 to D47 and D5 to D47, respectively (*p* ≤ 0.001), with both values reaching 0.00 ± 0.00 on D47. The high-dose group’s tumor volume and RTV were also significantly lower from D8 to D47 and D5 to D47 (*p* ≤ 0.001), with values of 95.62 ± 270.46 mm^3^ and 0.48 ± 1.36 on D47.

Similarly, the injectable paclitaxel DS micelle groups demonstrated remarkable, dose-dependent tumor inhibition. Specifically, the tumor volume and RTV in the middle-dose DS micelle group were significantly lower than those of the negative control group (*p* ≤ 0.001). Furthermore, when compared to the low-dose DS micelle group, its tumor volume was significantly lower from D12 to D47 (*p* ≤ 0.05, *p* ≤ 0.01, and *p* ≤ 0.001), and its mean RTV was also significantly reduced from D8 to D47.

#### 2.3.3. Relative Tumor Proliferation Rate (T/C%)

**Figure 7:**
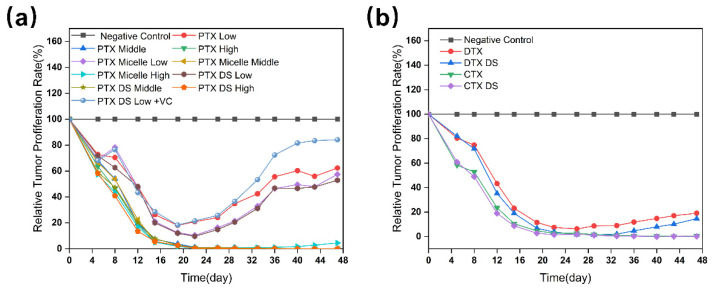
Relative tumor proliferation rate (T/C%) of MCF-7 tumor-bearing BALB/c nude mice following different treatments: (**a**) Paclitaxel formulations; (**b**) Docetaxel and cabazitaxel formulations.

As illustrated in Figure 7a, the T/C% for the low-dose albumin-bound paclitaxel group remained below 40% between D15 and D29, reaching its minimum value of 18.09% on D19. The T/C% of the middle-dose albumin-bound paclitaxel group stayed below 40% from D12 to D47, with a minimum of 0.03% on D47. For the high-dose albumin-bound paclitaxel group, the T/C% remained below 40% from D12 to D47, reaching its minimum of 0.00% between D43 and D47. In the injectable paclitaxel micelle groups, the low-dose group’s T/C% was below 40% from D15 to D33, with a minimum of 10.40% on D22; the middle-dose group remained below 40% from D12 to D47, with a minimum of 0.00% on D47; and the high-dose group was below 40% from D12 to D47, reaching its minimum of 0.45% on D22.

For the injectable paclitaxel DS micelle groups, the T/C% of the low-dose group remained below 40% from D15 to D33, with a minimum of 9.60% on D22. The T/C% of the middle-dose group was below 40% from D12 to D47, reaching a minimum of 0.02% on D47. The high-dose group’s T/C% remained below 40% from D12 to D47, reaching a minimum of 0.00% between D40 and D47. Additionally, the T/C% of the low-dose injectable paclitaxel DS micelle + VC group remained below 40% from D15 to D29, with a minimum of 18.39% on D19.

As shown in Figure 7b, the T/C% for the docetaxel injection group remained below 40% from D15 to D47, reaching its minimum of 6.30% on D26. The T/C% for the injectable docetaxel DS micelle group stayed below 40% from D12 to D47, with a minimum of 1.38% on D29. For the cabazitaxel injection group, the T/C% remained below 40% from D12 to D47, reaching a minimum of 0.13% on D43. The T/C% of the injectable cabazitaxel DS micelle group remained below 40% from D12 to D47, reaching a minimum of 0.00% on D40.

#### 2.3.4. Tumor Growth Inhibition Rate (IRTV%)

**Figure 8:**
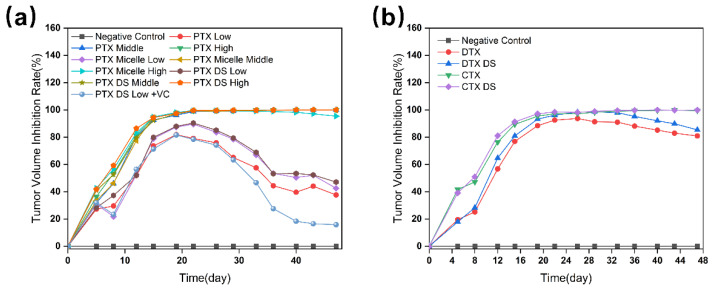
Tumor volume inhibition rate (IRTV%) profiles of MCF-7 tumor-bearing mice: (**a**) Paclitaxel formulations; (**b**) Docetaxel and cabazitaxel formulations.

According to Figure 8a, the tumor inhibition rate for the negative control group remained at 0% from D1 to D47. The tumor volume inhibition rate for the low-dose albumin-bound paclitaxel group peaked at 81.91% on D19, then declined to 37.66% on D47. The middle-dose group reached 92.64% on D15, remaining above 90% from D15 to D47 and exceeding 99% from D26 to D47. The high-dose group reached 94.58% on D15 and achieved 100% on D46 and D47. In the injectable paclitaxel micelle groups, the low-dose group peaked at 89.6% on D22 before decreasing to 42.51% on D47; the middle-dose group reached 94.53% on D15 and remained above 99% from D22 to D47; and the high-dose group reached 94.84% on D15, increasing to 100% by D47.

For the injectable paclitaxel DS micelle groups, the low-dose group peaked at 90.4% on D22, subsequently decreasing to 42.51% on D47. The middle-dose group rose to 92.14% on D15, maintaining over 99% from D19 to D47 and reaching 99.98% on D47. The high-dose group reached 94.34% on D15, remained above 99% from D22 to D36, and reached 100% from D40 to D47. The low-dose injectable paclitaxel DS micelle + VC group peaked at 81.64% on D19, but decreased to 15.82% by D47.

As shown in Figure 8b, the docetaxel injection group peaked at 93.7% on D26 and declined to 80.93% on D47. The docetaxel DS micelle group reached 93.26% on D19, peaked at 98.26% on D29, and declined to 85.36% on D47. The cabazitaxel injection group reached 95.4% on D19 and continued to increase, reaching 99.5% by D47. The injectable cabazitaxel DS micelle group reached 97.2% on D19 and increased to 99.85% by D47. Overall, DS micelles showed comparable or improved tumor-inhibitory effects in several treatment groups compared with the corresponding non-crosslinked micelles, and the observed in vivo performance is consistent with the antitumor potential reported for representative single-taxane redox-responsive micellar systems. However, because direct comparative release, IC_50_, biodistribution, and tumor-accumulation studies were not performed under identical conditions, the detailed mechanistic basis of the present system remains to be further clarified.

#### 2.3.5. Multi-Taxane Applicability of mPEG-PLA-(LA)_4_ DS Nanomicelles

A formulation-level advantage of the present mPEG-PLA-(LA)_4_ DS micelle system is its applicability to multiple taxane drugs. Representative redox-responsive taxane micelles reported previously commonly focus on one specific taxane per system. For example, Han et al. [35] reported CTX-loaded disulfide-crosslinked micelles based on lipoic-acid-grafted mPEG-PLA, Li et al. [36] developed reversible disulfide-crosslinked micelles for PTX delivery, and Koo et al. reported DTX-loaded core–shell-corona micelles with shell-specific redox-responsive cross-links [39]. In contrast, the present study applied the same structurally defined mPEG-PLA-(LA)_4_ carrier platform to three clinically relevant taxanes, namely PTX, DTX and CTX. This design demonstrates formulation versatility rather than single-drug applicability.

The in vivo antitumor results further support this multi-taxane applicability. For PTX-loaded DS micelles, the middle-dose group maintained an IRTV value above 99% from D19 to D47 and reached 99.98% on D47, while the high-dose group achieved 100% inhibition from D40 to D47. For DTX-loaded DS micelles, the maximum IRTV reached 98.26% on D29. For CTX-loaded DS micelles, the IRTV increased to 99.85% by D47. These results indicate that the antitumor performance of mPEG-PLA-(LA)_4_ DS micelles was not restricted to a single taxane formulation, supporting the potential of this carrier as a generalizable platform for hydrophobic taxane delivery. However, because PTX, DTX and CTX differ in intrinsic potency, pharmacokinetics and clinically tolerated doses, direct comparison among the three taxanes should be interpreted cautiously. The key in vivo outcomes supporting the multi-taxane applicability of mPEG-PLA-(LA)_4_ DS nanomicelles are summarized in Table 3.

#### 2.3.6. Mechanism of Enhanced Antitumor Efficacy

The dose-dependent antitumor efficacy of the DS micelles may be associated with the structural features of the mPEG-PLA-(LA)_4_ carrier. The nanoscale size and PEGylated surface of polymeric micelles have been reported to favor prolonged circulation and passive tumor accumulation through the enhanced permeability and retention (EPR) effect [40]. However, because biodistribution and tumor-accumulation experiments were not performed in the present study, passive tumor targeting was not directly demonstrated. Furthermore, disulfide-crosslinked micelles have been reported to improve micellar stability under non-reducing physiological conditions and reduce premature drug release. After cellular internalization, disulfide-containing nanocarriers may undergo reductive destabilization in the GSH-rich intracellular environment, as supported by previous studies showing GSH- or DTT-triggered disassembly and accelerated drug release from disulfide-crosslinked micellar systems [36,41,42]. Therefore, the antitumor efficacy observed in this study is consistent with, but does not directly prove, a literature-supported redox-responsive delivery mechanism. Direct GSH-triggered release or disassembly experiments under physiologically relevant reducing and non-reducing conditions should be included in future studies. In addition, although the in vivo efficacy results suggest that DS micelles may offer formulation advantages over non-crosslinked micelles, direct head-to-head comparisons of drug release, serum stability and tumor accumulation are still required to establish the mechanistic superiority of the crosslinked system. Because release kinetics under reducing and non-reducing conditions were not directly measured in the present study, the observed antitumor efficacy is not assigned to a specific GSH-triggered release mechanism.

### 2.4. Supportive Nonclinical Pharmacokinetic Evaluation of Paclitaxel Micelles

**Figure 9:**
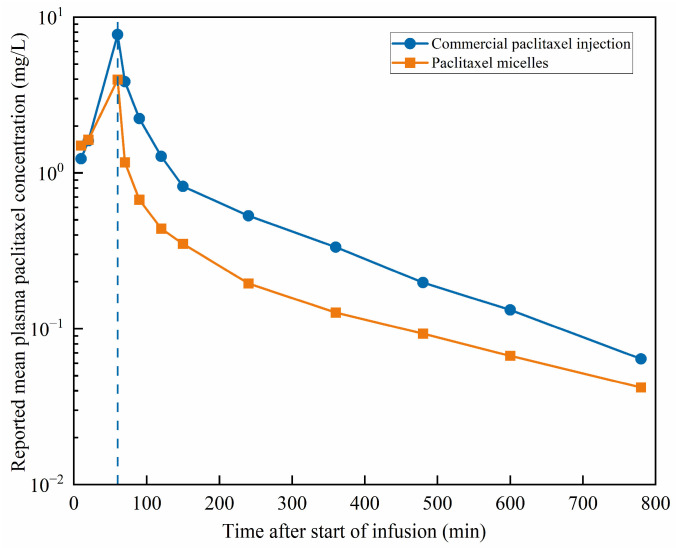
Mean plasma paclitaxel concentration–time profiles following a 60-min intravenous infusion of commercial paclitaxel injection or an injectable paclitaxel micelle formulation at 5 mg/kg in Beagle dogs using a randomized two-period crossover design (n = 8).

Supportive pharmacokinetic data were obtained from a randomized two-period crossover study in eight Beagle dogs following a 60-min intravenous infusion of paclitaxel at 5 mg/kg. As shown in Figure 9, the injectable paclitaxel micelle formulation produced a distinct plasma concentration–time profile compared with commercial paclitaxel injection.

The micellar formulation reduced AUC_0–∞_ from 609.87 ± 187.06 to 298.12 ± 49.35 mg·L^−1^·min and Cmax from 7.72 ± 1.44 to 3.97 ± 1.34 mg/L (*p* < 0.01). These results demonstrate that micellar formulation markedly alters the systemic disposition of paclitaxel.

## 3. Materials and Methods

### 3.1. Materials, Reagents, and Animals

The following chemical reagents were used in this study: dimethylolpropionic acid (DMPA), acetone, 2,2-dimethoxypropane, p-toluenesulfonic acid, dichloromethane (DCM), anhydrous magnesium sulfate, N,N’-dicyclohexylcarbodiimide (DCC), triethylamine (TEA), 4-pyrrolidinopyridine (4-py), dimethylolpropionic anhydride, lipoic acid neopentyl anhydride, diethyl ether, and ethanol. All chemicals were purchased from Sinopharm Chemical Reagent Co., Ltd. (Shanghai, China). Paclitaxel (PTX), docetaxel (DTX), and cabazitaxel (CTX) were purchased from Fujian Yewpark Biological Co., Ltd. (Sanming, China).

The primary laboratory equipment included an electronic analytical balance (BS224S, Sartorius AG, Göttingen, Germany), a rotary evaporator (RV10, IKA-Werke GmbH & Co. KG, Staufen, Germany), a diaphragm vacuum pump (GM-0.33B, Tianjin Jinteng, Tianjin, China), a constant temperature magnetic stirring bath (HWCC-5, Zhengzhou Greatwall, Zhengzhou, China), a low-temperature constant temperature reaction bath (DLSB-10/20, Shanghai Lingbiao, Shanghai, China), a vacuum oil pump (2XZ-2, Shanghai Vacuum Pump Factory, Shanghai, China), a vacuum drying oven (DZF-6020, Shanghai Hualian, Shanghai, China), an ultrasonic cleaner (SB-5200D, Ningbo Xinzhi, Ningbo, China), and an electric constant temperature water bath (DK-8D, Shanghai Jinghong, Shanghai, China).

The instruments used for structural and physicochemical characterization included an INVENIO S+ Fourier transform infrared spectrometer (Bruker, Ettlingen, Germany), an Ascend 600 nuclear magnetic resonance spectrometer operating at 600 MHz (Bruker, Rheinstetten, Germany), a LabRAM HR Raman spectrometer (Horiba, Palaiseau, France), a 752 UV–visible spectrophotometer (Shanghai Lichen Instrument Technology Co., Ltd., Shanghai, China), a Malvern Zetasizer instrument (Malvern Instruments, Malvern, UK) for particle size, PDI, and zeta-potential measurements, and a JEM-F200 field-emission transmission electron microscope (JEOL, Tokyo, Japan) for TEM observation.

A total of 160 female SPF-grade BALB/c nude mice (age: 4–5 weeks; weight: 10.9–15.9 g) were purchased from Beijing Tonglihua Experimental Animal Co., Ltd. (Beijing, China). After tumor establishment, 120 mice were selected and randomly assigned to treatment groups when they reached 5–6 weeks of age. All animal experiments were approved by the Institutional Animal Care and Use Committee of East China University of Science and Technology under approval number ECUST-20252-088, and were conducted in accordance with institutional guidelines for the care and use of laboratory animals.

### 3.2. Synthesis of mPEG-PLA-(LA)_4_ Copolymer

#### 3.2.1. Synthesis of Hydroxyl-Protected Dimethylolpropionic Anhydride

DMPA was dissolved in acetone, followed by the addition of 2,2-dimethoxypropane and p-toluenesulfonic acid. Upon completion of the reaction, the solvent was evaporated. The product was re-dissolved in DCM, washed twice with water, and the organic layer was dried over anhydrous magnesium sulfate. After filtration and evaporation, hydroxyl-protected DMPA was obtained. This product was then dissolved in DCM, and DCC was added as a coupling agent. After the reaction, the mixture was filtered, and the filtrate was evaporated. The residue was dissolved in n-hexane, and the final protected DMPA anhydride was obtained via cold precipitation, filtration, and drying.

#### 3.2.2. Synthesis of mPEG-PLA-Ac

20 g of mPEG-PLA, 125 mL of DCM, 3.5 mL of TEA, 0.356 g of 4-py, and 7.85 g of DMPA anhydride were reacted in a round-bottom flask at room temperature for 24 h. DCM was removed by rotary evaporation at 45 °C. The residue was dissolved in anhydrous ethanol and recrystallized at −10 °C. The product was collected by suction filtration, and this purification step was repeated three times.

#### 3.2.3. Synthesis of mPEG-PLA-(OH)_4_

mPEG-PLA-Ac was dissolved in methanol and reacted with Dowex H^+^ ion exchange resin for 24 h. After filtration, the product was precipitated in diethyl ether and vacuum dried to obtain mPEG-PLA-(OH)_2_. Subsequently, mPEG-PLA-(OH)_2_ was reacted with DMPA anhydride in DCM using TEA and 4-py as catalysts for 6 h. The product, mPEG-PLA-(Ac)_2_, was purified by ethanol recrystallization and drying. Finally, mPEG-PLA-(Ac)_2_ was deprotected using Dowex H^+^ resin in methanol at 25 °C for 24 h. The solution was filtered, precipitated in ice-cold diethyl ether, and dried to yield the target mPEG-PLA-(OH)_4_.

#### 3.2.4. Synthesis of Lipoic Acid Anhydride

17.1 g of lipoic acid, 12.51 mL of TEA, and 10.5 mL of TMAC were dissolved in approximately 260 mL of ethyl acetate. The reaction was maintained at 0 °C for 2 h and then at room temperature for 1 h. The mixture was filtered through a G4 funnel. The filtrate was evaporated at 35 °C to remove ethyl acetate and then re-dissolved in DCM.

#### 3.2.5. Synthesis of mPEG-PLA-(LA)_4_

mPEG-PLA-(OH)_4_. was dissolved in DCM and added to a flask containing the synthesized lipoic acid anhydride, 12 mL of TEA, and 1.23 g of 4-py. The mixture was stirred at room temperature for 24 h. After DCM removal at 45 °C, the product was purified by repeated anhydrous ethanol recrystallization (four times) and finally vacuum dried.

#### 3.2.6. Structural Characterization of mPEG-PLA-(LA)_4_

The chemical structure of mPEG-PLA-(LA)_4_ was characterized by FT-IR, Raman spectroscopy, and ^1^H-NMR. FT-IR spectra of mPEG-PLA and mPEG-PLA-(LA)_4_. were recorded using an INVENIO S+ Fourier transform infrared spectrometer (Bruker, Germany) over the range of 4000–500 cm^−1^. Raman spectra were recorded using a LabRAM HR Raman spectrometer (Horiba, France) with a 785 nm excitation laser. The spectra were collected over the range of 400–700 cm^−1^, with particular attention to the 500–550 cm^−1^ region corresponding to the S–S stretching vibration. ^1^H-NMR spectra were recorded on an Ascend 600 nuclear magnetic resonance spectrometer (Bruker, Germany) operating at 600 MHz, using CDCl_3_ as the solvent.

### 3.3. Preparation of CTX-Loaded Nanomicelles (10% TDL)

To prepare CTX-loaded micelles, 20 mg of CTX and 180 mg of mPEG-PLA-(LA)_4_ were dissolved in ethyl acetate. A thin drug-polymer film was formed via rotary evaporation at 45 °C and subsequently hydrated with deionized water to form a self-assembled CTX solution. Under a nitrogen atmosphere, the pH was adjusted to 7.5–8.0 using ammonia water. Then, 6 mg of dithiothreitol (DTT) was added to transiently reduce/open the lipoic-acid-derived cyclic disulfide moieties, and the mixture was stirred for 15 min. The solution was dialyzed overnight using a dialysis membrane with a molecular weight cutoff of 8000–14,000 Da to remove excess DTT. After dialysis, thiol groups were allowed to re-oxidize to form disulfide-crosslinked nanomicelles. The final CTX-loaded nanomicelles were obtained by filtration through a 0.22 μm membrane.

### 3.4. Physicochemical Characterization of CTX-Loaded Nanomicelles

The hydrodynamic diameter, polydispersity index (PDI), and zeta potential of CTX-loaded disulfide-crosslinked nanomicelles prepared at different theoretical drug loading ratios were measured using a Malvern Zetasizer instrument (Malvern Instruments, UK). Before measurement, the micellar suspension was diluted with deionized water to an appropriate concentration. All measurements were performed at 25 °C in triplicate, and the results are expressed as mean ± standard deviation (SD).

The morphology of CTX-loaded disulfide-crosslinked nanomicelles was observed using a JEM-F200 field-emission transmission electron microscope (JEOL, Japan). Briefly, a representative CTX-loaded micellar suspension prepared at 6.0% theoretical drug loading was diluted with deionized water to 0.1 mg/mL and dropped onto a carbon-coated copper grid. After standing for 1–2 min, excess liquid was removed with filter paper. The grid was then negatively stained with phosphotungstic acid solution for 15 min, and the excess staining solution was gently removed with filter paper. The sample was dried at room temperature before TEM observation.

The apparent encapsulation efficiency of CTX-loaded mPEG-PLA-(LA)_4_ DS nanomicelles was determined using a centrifugation–UV spectrophotometric method. The freshly prepared nanomicellar suspension was centrifuged at 12,000× *g* for 20 min at 25 °C. Subsequently, 200 μL of the supernatant was collected and diluted with ethanol to a final volume of 10.0 mL.

A matched LA4 blank containing the same carrier concentration was processed under identical conditions to correct for carrier-derived absorption. The absorbance was measured at 228 nm, and the CTX concentration was determined using the established calibration curve. The apparent encapsulation efficiency was calculated as the ratio of the carrier-corrected CTX concentration in the post-centrifugation supernatant to the theoretical CTX concentration after dilution.

### 3.5. In Vivo Animal Experiments

#### 3.5.1. Establishment of Tumor Models

Human breast cancer MCF-7 tumor fragments (~2 mm^3^) were subcutaneously inoculated into the right axillary region of female BALB/c nude mice. BALB/c nude mice were selected because their impaired T-cell-mediated immunity allows the survival and serial passage of human tumor xenografts, making them suitable for antitumor efficacy evaluation. No exogenous estrogen supplementation was administered during tumor establishment or treatment. To ensure tumor uniformity, seed mice were used for serial passage. After aseptic removal, tumor tissues were cut into fragments and re-inoculated into the mice for formal experimentation.

Mice with tumor volumes between 102.23 and 264.28 mm^3^ were selected for grouping. Animals with failed tumor establishment, tumor ulceration before grouping, abnormal general condition, or tumor volumes outside the predefined enrollment range were excluded before randomization. Eligible mice were randomly assigned to treatment groups using a random-number table while balancing the baseline tumor-volume distribution among groups.

#### 3.5.2. Experimental Procedures and Dosing Regimens

All therapeutic formulations in this study were administered via tail vein injection. The determination of dosing levels was guided by the FDA’s “Guidelines for the Conversion of Equivalent Doses Between Animals and Humans” [43] and further optimized based on preliminary maximum tolerated dose (MTD) pre-experiments. Specifically, paclitaxel (PTX) was administered in three dose gradients: low (15 mg/kg), medium (30 mg/kg), and high (50 mg/kg); the dose for docetaxel (DTX) was set at 10 mg/kg. The administration schedule for different taxanes was strictly designed according to their respective pharmacokinetic profiles. Given the higher clearance rates of PTX and DTX in mice, a dense dosing regimen of once every 4 days for a total of 3 injections was utilized. Conversely, the administration frequency for cabazitaxel (CTX) was set to once every 3 weeks for a total of 2 doses, referencing its clinical standard protocols and its terminal half-life of approximately 95 h [23,44], which aimed to circumvent lethal cumulative toxicity and simulate real-world clinical characteristics. Furthermore, to investigate potential antagonistic mechanisms, a combination treatment group was established, wherein mice received an additional dose of sodium vitamin C (474 mg/kg) via tail vein 8 h after the injection of the low-dose PTX DS micelle. All treatment groups were compared in parallel with equivalent doses of commercially available free drug formulations to evaluate the in vivo antitumor efficacy and preliminary tolerability of the nanomicelles.

#### 3.5.3. Experimental Grouping and Administration Details

Tumor-bearing mice were randomly assigned into 15 experimental groups (n = 8 per group) based on tumor volume, including a negative control group, albumin-bound PTX at low (15 mg/kg), medium (30 mg/kg), and high (50 mg/kg) doses, DTX injection (10 mg/kg), injectable DTX DS (disulfide-crosslinked) micelles (10 mg/kg), CTX injection (15 mg/kg), and injectable CTX DS micelles (15 mg/kg). Additional groups included injectable PTX micelles at low (15 mg/kg), medium (30 mg/kg), and high (50 mg/kg) doses, injectable PTX DS micelles at low (15 mg/kg), medium (30 mg/kg), and high (50 mg/kg) doses, and a combination group of low-dose injectable PTX DS micelle plus sodium vitamin C (15 + 474 mg/kg). All groups received tail vein injections with a dosing volume of 10 mL/kg, except for the high-dose PTX DS micelle group (Group 14), which received 12.1 mL/kg. For the combination group (Group 15), sodium vitamin C was administered 8 h post-injection of the PTX DS micelle, while the negative control group received an equivalent volume of the vehicle.

#### 3.5.4. Clinical Observation and Efficacy Evaluation

General clinical observations were conducted twice daily following the initial administration. Body weight and tumor dimensions (long axis L and short axis W) were measured twice weekly using a vernier caliper. Humane endpoints were predefined as tumor volume exceeding 2000 mm^3^, body-weight loss greater than 20%, tumor ulceration or necrosis, severe impaired mobility, moribund state, or obvious signs of severe distress. During the experimental period, no animals reached the predefined humane endpoints, and no animals were euthanized before the scheduled study end point. Tumor volume and body weight were measured by investigators who were not blinded to treatment allocation. The observation endpoints were as follows: D48 for the negative control, low-dose albumin-bound PTX, low-dose injectable PTX micelle, low-dose injectable PTX DS micelle, and Group 15; D68 for DTX groups; D76 for CTX groups; and D92 for medium- and high-dose PTX groups.

Tumor volume (V) was calculated using the following formula:

$$V=\frac{1}{2}\times L\times W^{2}$$

Therapeutic efficacy was evaluated using the following parameters:

Relative Tumor Volume (RTV):

$$RTV=\frac{V_{t}}{V_{0}}$$

where $V_{t}$ is the tumor volume at the time of measurement and $V_{0}$ is the tumor volume at the first administration.

Relative Tumor Proliferation Rate (T/C%):

$$\frac{T}{C}(\%)=\frac{Mean\;RTV\;of\;treated\;group}{Mean\;RTV\;of\;control\;group}\times 100\%$$

Tumor Volume Inhibition Rate (IRTV%):

IR(%)=100%−T/C

### 3.6. Supportive Nonclinical Pharmacokinetic Study

Eight Beagle dogs received an injectable paclitaxel micelle formulation and commercial paclitaxel injection at 5 mg/kg by intravenous infusion over 60 min using a randomized two-period crossover design with a 14-day washout period. Blood samples were collected before administration and at 10, 20, 60, 70, 90, 120, 150, 240, 360, 480, 600, and 780 min after the start of infusion.

Plasma paclitaxel concentrations were determined by HPLC using a C18 column and UV detection at 232 nm. Cmax was obtained from the measured concentrations, AUC was calculated using the trapezoidal method, and the two formulations were compared using a paired *t*-test.

### 3.7. Statistical Analysis

Statistical analyses were performed using SPSS 13.0 software. Quantitative data are presented as mean ± standard deviation (SD). For repeated measurements of body weight, tumor volume, relative tumor volume (RTV), and tumor volume inhibition rate (IRTV%) over time, statistical comparisons among groups were performed at each time point. Levene’s test was first used to assess homogeneity of variance. If the variance was homogeneous, one-way analysis of variance (ANOVA) followed by the LSD post hoc test was performed. If the variance was not homogeneous, the Kruskal–Wallis test was used, followed by the Mann–Whitney U test for pairwise comparisons when appropriate. A value of *p* < 0.05 was considered statistically significant. After D48, due to animal death in the negative control group, the corresponding data were presented as mean ± SD only and were not included in statistical comparisons.

## 4. Study Limitations

This study still has several limitations. The present work mainly evaluated the in vivo antitumor efficacy of taxane-loaded mPEG-PLA-(LA)_4_ DS nanomicelles in the MCF-7 subcutaneous xenograft model. Some important issues were not examined in this study, including in vitro antitumor mechanism, redox-triggered drug release, biodistribution, tumor accumulation, and long-term safety. Supportive pharmacokinetic data were available for a paclitaxel micelle formulation, whereas comparative pharmacokinetic studies of the three present taxane formulations remain to be completed. In particular, the contribution of the EPR effect was not directly measured, and the influence of the ultra-small micelle size on blood circulation, renal clearance, and tissue distribution remains unclear. In addition, the safety evaluation was mainly based on body-weight monitoring, so hematological analysis, serum biochemical tests, histopathological examination, and long-term toxicity studies are still needed.

Another limitation is that only one breast cancer model was used. Since breast cancer includes different molecular subtypes and tumor microenvironments, more models, such as HER2-positive, triple-negative, orthotopic, or metastatic breast cancer models, should be included in future studies. Moreover, the comparison with previously reported redox-responsive taxane nanocarriers was mainly based on published literature. Because these studies differ in carrier structure, drug type, tumor model, dose, and evaluation method, the current comparison should be regarded as a literature-based discussion rather than direct proof of superiority. Parallel head-to-head studies will be needed to better clarify the relative advantages of the present mPEG-PLA-(LA)_4_ DS micelle system.

## 5. Conclusions

In this study, an mPEG-PLA-(LA)_4_ block copolymer was successfully synthesized and used to prepare disulfide-crosslinked (DS) nanomicelles for the delivery of taxanes, including paclitaxel (PTX), docetaxel (DTX), and cabazitaxel (CTX). Structural characterization by FT-IR, Raman spectroscopy, and ^1^H-NMR supported the successful introduction of LA-derived disulfide-containing structures into the mPEG-PLA copolymer. The prepared CTX-loaded DS nanomicelles showed nanoscale particle size, acceptable dispersity, and near-neutral/slightly negative zeta potential, supporting their formation as nanosized micellar assemblies.

In vivo antitumor evaluation in MCF-7 tumor-bearing mice showed that the taxane-loaded DS micelles produced marked tumor-growth inhibition, particularly in the middle- and high-dose PTX DS micelle groups and the CTX DS micelle group. Body-weight monitoring suggested preliminary gross tolerability during the treatment period. Supportive pharmacokinetic results further showed that micellar formulation markedly reduced the peak plasma concentration and systemic plasma exposure of paclitaxel relative to commercial injection. However, because comprehensive toxicological evaluations, including hematology, serum biochemistry, and histopathological analysis, were not performed, systemic safety cannot be concluded from the present data alone.

Overall, these results suggest that mPEG-PLA-(LA)_4_ DS nanomicelles may serve as a potential carrier platform for hydrophobic taxane delivery in breast cancer therapy. Nevertheless, active targeting, biodistribution, tumor accumulation, comprehensive pharmacokinetic/pharmacodynamic relationships for the three taxane formulations, direct GSH-triggered release behavior, batch-to-batch reproducibility, scalability, and long-term safety remain to be further investigated before clinical translation can be considered.

Beyond carrier synthesis, the present work demonstrates the multi-taxane applicability of mPEG-PLA-(LA)_4_ DS micelles. The same carrier platform was applied to PTX, DTX and CTX, and all three taxane-loaded DS micelles produced pronounced antitumor activity in the MCF-7 xenograft model. This formulation versatility distinguishes the present system from many reported redox-responsive micelles that focus on a single taxane formulation.

## Figures and Tables

**Table 1:** Physicochemical properties of CTX-loaded disulfide-crosslinked nanomicelles prepared at different theoretical drug loading ratios. Data are expressed as mean ± SD, *n* = 3.

Theoretical Drug Loading (%)	Particle Size (nm)	PDI	Zeta Potential (mV)
6	10.15 ± 0.02	0.194 ± 0.001	−3.73 ± 0.56
8	12.36 ± 0.03	0.158 ± 0.011	0.06 ± 0.52
10	12.75 ± 0.02	0.211 ± 0.004	−3.51 ± 1.06
12	16.57 ± 0.03	0.460 ± 0.008	ND

ND: Not detected.

**Table 2:** Particle-size benchmarking of the present mPEG-PLA-(LA)_4_ DS micelles with representative disulfide-crosslinked taxane micellar systems.

Carrier System	Structural Feature	Drug	Particle Size (nm)	Ref.
mPEG-PLA-(LA)_4_ DS micelles	Four predetermined LA sites	CTX	12.75 ± 0.02	This work
LA-grafted mPEG-PLA DCL micelles	LA-grafted mPEG-PLA; disulfide core-crosslinking	CTX	18.97 ± 0.23	Han et al. [35]
Reversible disulfide-crosslinked micelles	Disulfide core-crosslinked micelles	PTX	~28 nm	Li et al. [36]

**Table 3:** Summary of the multi-taxane applicability of mPEG-PLA-(LA)_4_ DS nanomicelles in the MCF-7 xenograft model.

Taxane	Structural Feature Micelle Formulation	Key In Vivo Outcome	Interpretation
PTX	PTX-loaded mPEG-PLA-(LA)_4_ DS micelles	Middle dose: IRTV 99.98% on D47; high dose: IRTV 100% from D40 to D47	Strong dose-dependent tumor inhibition
DTX	mPEG-PLA-(LA)_4_ DS micelles	Peak IRTV 98.26% on D29; D47 IRTV 85.36%	Effective tumor inhibition, with later partial tumor regrowth
CTX	CTX-loaded mPEG-PLA-(LA)_4_ DS micelles	IRTV 99.85% on D47	Sustained strong tumor inhibition

## Data Availability

The original contributions presented in this study are included in the article. Further inquiries can be directed to the corresponding authors.

## Abbreviations

The following abbreviations are used in this manuscript:

mPEG	Poly(ethylene glycol) methyl ether
PLA	Poly(lactic acid)
PTX	Paclitaxel
DTX	Docetaxel
CTX	Cabazitaxel
DS	Disulfide-crosslinked
DMPA	Dimethylolpropionic acid
GSH	Glutathione
EPR	Enhanced permeability and retention effect
DLS	Dynamic light scattering
PDI	Polydispersity index
TDL	Theoretical drug loading
RTV	Relative tumor volume
DTT	Dithiothreitol
DCM	Dichloromethane
TEA	Triethylamine
4-py	4-pyrrolidinopyridine
DCC	N,N’-dicyclohexylcarbodiimide
IRTV	Tumor volume inhibition rate
MTD	Maximum tolerated dose
